# Epitranscriptomic mechanisms and implications of RNA m^5^C modification in cancer

**DOI:** 10.7150/thno.112332

**Published:** 2025-07-25

**Authors:** Zhonghao Mao, Yan Tian, Lisha Wu, Yu Zhang

**Affiliations:** 1 Department of Gynecology, Xiangya Hospital, Central South University, Changsha, China.; 2 Gynecological Oncology Research and Engineering Center of Hunan Province, Changsha, China.; 3 National Clinical Research Center for Geriatric Disorders, Xiangya Hospital, Central South University, Changsha, China.; 4 Institute of Medical Sciences, Xiangya Hospital, Central South University, Changsha, China.

**Keywords:** 5-methylcytidine modification, RNA-modifying proteins, cancer, clinical application

## Abstract

Cancer is an extremely complex disease characterized by abnormal cell growth due to genetic and environmental factors. With the rise of the field of epigenetic transcriptomics, 5-methylcytidine (m^5^C) modification has been identified as one of the most common chemical modifications occurring in various RNA types. The writers, erasers, and readers of m^5^C modification regulate cancer initiation, progression, and therapeutic responses, such as the proliferation, metastasis, angiogenesis, metabolic reprogramming, immune escape, and therapeutic resistance of tumour cells, by regulating RNA stability, translation, nuclear export, and splicing processes. In this review, we elucidate the biological process of m^5^C modification, summarize the abnormal expression of RNA-modifying proteins (RMPs) in common malignant tumours, explore their functional effects on malignant hallmarks of cancer and molecular mechanisms, and prospect the potential clinical application value of m^5^C.

## 1. Introduction

Cancer has become a major public health challenge that threatens human health worldwide. More than 52,900 people are diagnosed with cancer every day, and more than 27,000 people die from it, which places a serious economic burden on society 1. The initiation of cancer was originally thought to be an entirely genetic disease driven by genes. However, the complexity of cancer reveals that it is a highly structured ecosystem that controls tumour initiation, progression, and therapeutic response 2.

Recently, the discovery of reversible mRNA methylation has opened a new scope of posttranscriptional gene regulation in eukaryotes, especially its role in cancer initiation, progression, and treatment 3-5. More than 170 RNA modifications have been identified in eukaryotes 6. Among these modifications, 5-methylcytidine (m^5^C) RNA modification has attracted increasing attention in cancer research 7. Initially, m^5^C modification sites were found mainly in tRNA and rRNA. In 2012, bisulphite sequencing (BisSeq) of whole transcripts in HeLa cells revealed that m^5^C modification was widely distributed in mRNA and noncoding RNA (ncRNA), and the first transcriptome-wide mapping of m^5^C in human cells was performed 8. The roles of TET and ALYREF in m^5^C were subsequently identified in 2014 and 2017, respectively 9, 10. An increasing number of studies have shown that m^5^C is involved in various diseases, such as cardiovascular, liver, Alzheimer, SARS-CoV-2-associated, and autoimmune disease, as well as several cancers 11-14. Previous excellent reviews have highlighted progress in understanding the role of RNA m^5^C modification in multiple diseases 13, 15-19. In addition to discussing newly discovered RNA-modifying proteins (RMPs), m^5^C modification target RNAs, and updates in our understanding of RNA m^5^C modification mechanisms and functions in cancers, we summarize the comprehensive functions and molecular mechanisms of RNA m^5^C modification in more than twenty malignant tumours according to cancer initiation, progression, and therapeutic response. Importantly, we fill gaps in the study of several RMPs in specific cancers, which may provide new ideas for discovering potential biomarkers and therapeutic targets. We also propose the clinical application potential of m^5^C modification, which lays the foundation for further research.

## 2. m^5^C RNA Modification

**Figure 1:**
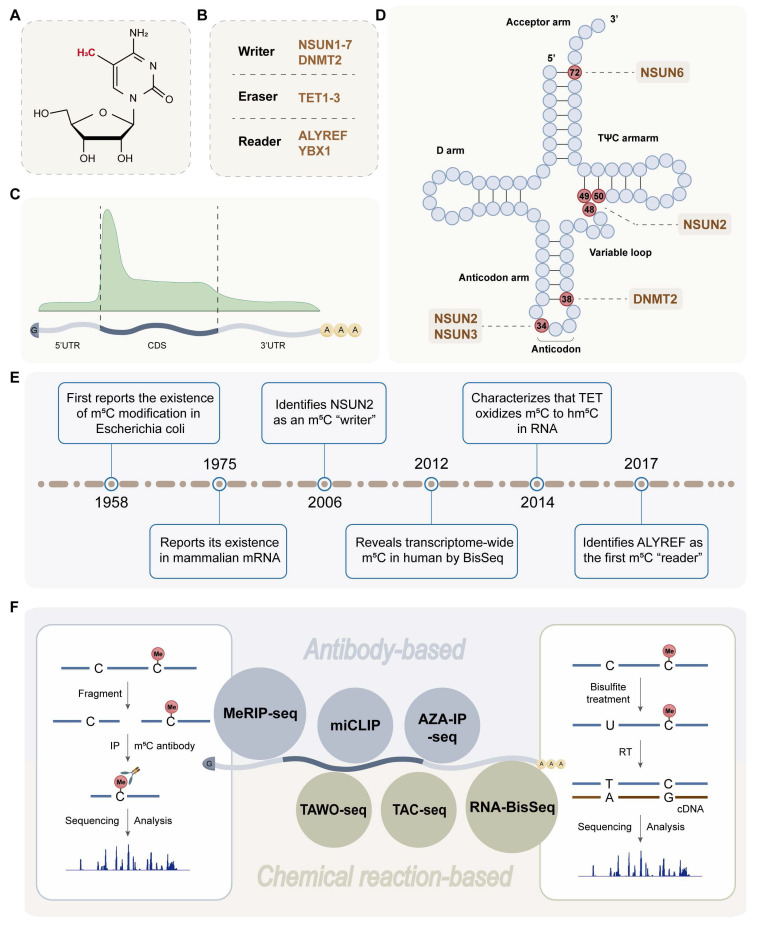
The molecular structure (A), RNA-modifying proteins (B), sites distribution on mRNA (C) and tRNA (D), development history(E) and detection techniques (F) of m^5^C modification. Created in BioRender. Mao, Z. (2025) https://BioRender.com/6nefran.

**Figure 2:**
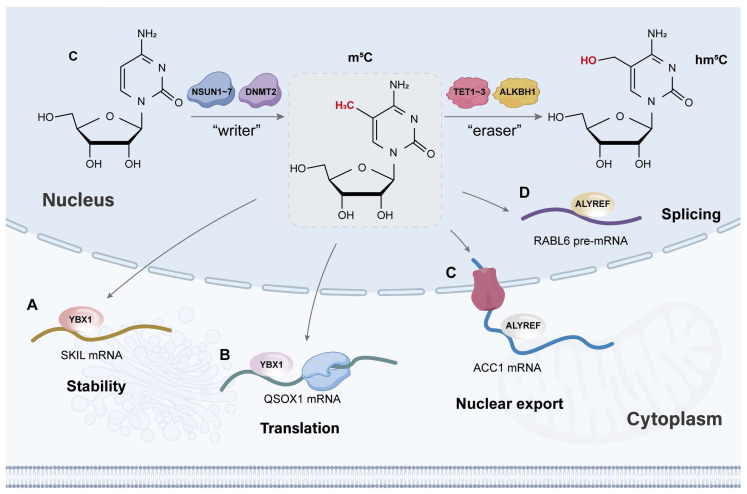
The biological processes and molecular mechanisms of m^5^C function in tumour cells. (A)NSUN2/YBX1/m^5^C regulates SKIL stability in colorectal cancer. (B)NSUN2/YBX1/m^5^C regulates QSOX1 translation in lung cancer. (C)NSUN5/ALYREF/m^5^C regulates ACC1 nuclear export in prostate cancer. (D)NSUN2/ALYREF/m^5^C regulates RABL6 in bladder cancer. Created in BioRender. Mao, Z. (2025) https://BioRender.com/n3el9cc.

**Figure 3:**
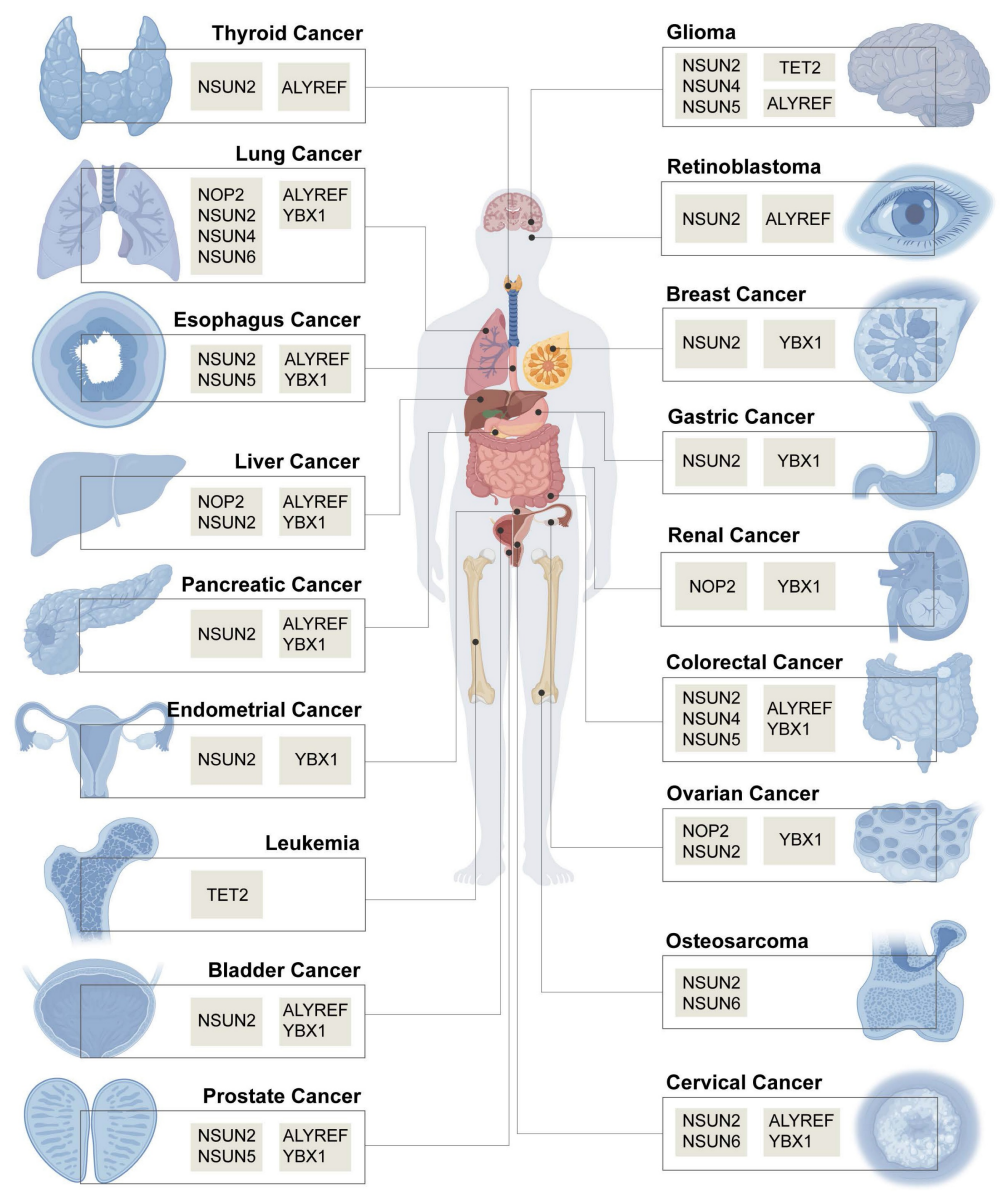
The m^5^C RNA-modifying proteins that play a key role in cancer. Created in BioRender. Mao, Z. (2025) https://BioRender.com/eu8anj3.

m^5^C is a chemical modification formed by the addition of a methyl group from the donor, usually S-adenosyl-methionine (SAM), to the fifth carbon atom of cytosine in the RNA molecule 20 (Figure 1A-B). In mammals, m^5^C modification accounts for approximately 0.02-0.09% of all cytosine modifications 21. The first cytosine-methylated transcriptome analysis of human cells revealed more than 10,000 m^5^C sites (>20% methylation) located on approximately 8,500 mRNAs 22, mainly distributed in the coding sequence (CDS) region 23 (Figure 1C). Since m^5^C was first reported in 1958, this modification has been found to occur in any RNA type, including mRNA, tRNA (Figure 1D), rRNA, and eRNA, among others 17. Owing to the limitations of m^5^C modification in coding RNA research, Squires *et al.* innovatively combined sulphite cell RNA transformation with next-generation sequencing in 2012 8 (Figure 1E), which promoted our understanding and further exploration of m^5^C modification in mRNA. Recently, various methods of m^5^C detection have emerged 24, 25. There are two main categories, namely, antibody-based and chemical reaction-based methods (Figure 1F). Methylated RNA immunoprecipitation sequencing (MeRIP-seq) uses antibodies specific for m^5^C or m^5^C methyltransferase to enrich m^5^C-modified RNA 26. Currently, RNA-BisSeq is the most widely used strategy for transcriptome-wide, base-resolution m^5^C detection 27, 28.

m^5^C modification is dynamically regulated by three types of RMPs, namely, writers, erasers, and readers 29 (Figure 2). Among them, the main writers include NOL1/NOP2/SUN (NSUN) domain protein family members 30-37 and DNA methyltransferase (DNMT) homologous DNMT2 38, 39, and the erasers include the TET family (TET1-3) 9, 40, 41 and alkB homologue 1 (ALKBH1) 42, 43. The m^5^C sites in RNA are recognized by two main readers, Aly/REF export factor (ALYREF) 10, 44, 45 and Y-box binding protein 1 (YBX1) 46-48, which determine the regulatory mechanism and function of m^5^C modification in tumour cells. To date, the dysregulated RMPs that have been studied in the field of oncology are shown in Figure 3.

### 2.1 Writers

m^5^C RNA methyltransferases (RNMTs) first form a covalent thioester bond, connecting the cysteine residue of their catalytic domain to the C6 position of the target cytosine, forming an RNMT-RNA adduct 49. Then, RNMTs catalyse the transfer of a methyl group from SAM to the fifth carbon of the cytosine base, forming m^5^C 50. NSUN1-7 and DNMT2, as RNMTs, catalyse the m^5^C modification on different RNAs in different subcellular locations, thereby exerting their respective biological functions.

The human NSUN2 gene is located at 5p15, and its protein has been shown to be localized to the nucleoli situated between or in close proximity to dense heterochromatic regions 51. The role of NSUN2 is quite extensive, as it acts on a variety of RNA types, such as tRNA, mRNA, and ncRNA. NSUN2-mediated m^5^C modification of tRNA is common and highly conserved, occurring in the vast majority (>80%) of transcribed tRNA *in vivo* in humans and mice 52. Moreover, recognition and methylation by NSUN2 are both site- and structure specific. tRNA contains five conserved domains, including the acceptor arm, the D arm, the anticodon arm, the variable loop (VL) and the TΨC arm (Figure 1D). For eukaryotic tRNA, m^5^C residues cluster at the junction between the VL and TΨC arms, and C48 and C49 are most frequently modified, with a high prevalence 52. BisSeq and miCLIP have confirmed robust m^5^C modification in the anticodon loop at C34 (tRNA^Leu^) and C38 (tRNA^Asp^, tRNA^Gly^, and tRNA^Val^) and in the VL junction at C50 (tRNA^Glu^ and tRNA^Gly^) 52, 53. Notably, methylation at C34/48/49/50 is solely dependent on NSUN2, whereas C38 methylation is mediated by DNMT2 32, 39, 54. Mechanistically, NSUN2 protein accommodates the SAM cofactor with its Rossmann-fold catalytic core (residues 171-429) and PUA domain (residues 54-147). In addition, it uses two catalytic cysteines in the active site, which are present in conserved motifs IV (Cys271) and VI (Cys321) 55. The deposition of m^5^C at the VL protects tRNA from tRNA‒protein interactions and unnecessary cleavage of mature and functional tRNA during the stress response 55. In contrast, the functional loss of NSUN2 could result in the absence of tRNA Leu^CAA^ and lead to changes in codon usage, significantly impacting the translation rate of tissue-specific proteins in mammals 56. NSUN2 is the most important RNA methyltransferase to induce m^5^C to specific RNAs that regulate the malignant behaviour of various cancers 57-60. As a cofactor, glucose binds to NSUN2 at amino acids 1-28 to promote NSUN2 oligomerization and activation. Activated NSUN2 increases m^5^C methylation on *TREX2* mRNA and stabilizes* TREX2* to restrict cytosolic dsDNA accumulation and cGAS/STING activation to promote tumorigenesis and resistance to anti-PD-L1 immunotherapy 61.

NSUN1 (NOP2) is characterized primarily in budding yeast as an essential ribosomal biogenesis factor required for the deposition of m^5^C on 25S rRNA 31. miCLIP-seq has revealed that rRNA is the major m^5^C-specific target of NSUN1 in human cells. Human NSUN1 binds to the rRNA 5′-ETS region and crosslinks to 28S rRNA at position C4447 62. NSUN3 initiates m^5^C biogenesis at position C34 in human mitochondrial tRNA^Met^, regulating mitochondrial protein synthesis, oxygen consumption, and mitochondrial activity 33. Mitochondrial m^5^C modification is essential for the dynamic regulation of mitochondrial translation rates and thereby shapes metabolic reprogramming during tumour metastasis 63. NSUN4 methylates cytosine 911 in the 12S rRNA of the small subunit (SSU), playing a key role in controlling the final step of ribosomal biogenesis to ensure that only the mature SSU and large subunit (LSU) are assembled 34, 64. Similarly, NSUN5 acts as an RNA methyltransferase at the C3782 position of human 28S rRNA, which regulates the adaptive translational program for survival under conditions of cellular stress 65. Human NSUN6 is associated with tRNA and acts as a tRNA methyltransferase. It can catalyse cytosine 72 at the 3' end of tRNA^Cys^ and tRNA^Thr^ 66.

Although the sequence and structure of DNMT2 (also known as TRNMT1) have close affinities for authentic DNA cytosine methyltransferases, the substrate of the highly conserved human DNMT2 was found to be predominantly aspartic acid-transfer RNA because the presence of a DNA competitor weakens but cannot eliminate the DNMT2-RNA complex signal 39, 67. An increasing number of studies have shown that DNMT2 and its homologues can modify C38 of tRNA^Gly^, tRNA^Asp^, tRNA^Glu^, and tRNA^Val^ *in vivo* in mammals and other species 68-71. In contrast with the NSNU family, only DNMT2 methylates RNA by utilizing a single cysteine (Cys79) in its catalytic pocket and through a DNMT-like catalytic mechanism 72. tRNA methylation catalysis by human DNMT requires C79 in motif IV (PCQ), E119 in motif VI (ENV), and R160 and R162 in motif VIII (RXR). In addition, some residues (such as I228, Q221, L229, G305, and Y297) located on the surface of the target recognition domain (TRD) and target recognition extension domain (TRED) regions in DNMT2 contribute to the selection of preferred substrate tRNA 72, 73. Importantly, DNMT and NSUN2 exhibit complementary target site specificities and collaborate to facilitate tRNA methylation by complementing each other in terms of gene expression, promoting tRNA stability and accurate protein synthesis 68, 74-76. DNMT2 is widely involved in a variety of physiological regulatory processes. For example, DNMT2 deletion increases cancer cell sensitivity to radiation and PARP inhibitors (PARPis). This role is dependent on its m^5^C writer effect 77. DNMT2-dependent m^5^C in damage-induced R-loops promotes transcription coupled-homologous recombination (TC-HR) and simultaneously suppresses PARP1-mediated alternative nonhomologous end joining (Alt-NHEJ), ensuring that TC-HR is the preferred double-strand break (DSB) repair pathway in transcribed regions 78. Overall, previous studies have shown that the above RNMTs function primarily on tRNA and rRNA. Interestingly, they have been shown to be associated with mRNA methylation 79.

### 2.2 Erasers

As a dynamic process, added methyl groups can be removed by demethylases (erasers). Previously, the reversible biological process of m^5^C modification has remained controversial. Over the years, m^5^C has been shown to be oxidized by TET1-3 and ALKBH1 to bioactive 5-hydroxymethylcytosine (hm5c) 9, 42, 63, 80.

TET family members were initially identified as DNA demethylases for a variety of nucleic acid substrates 81. The primary structure of TET enzymes includes a carboxy-terminal catalytic domain composed of a cysteine-rich domain (CRD) and two double-stranded β-helix (DSBH) regions flanking an extended low-complexity insertion region 82. The DSBH domains harbour conserved residues critical for coordinating cofactors (Fe(II) and α-ketoglutarate) essential for catalysis. The catalytic core is stabilized by two zinc finger motifs that structurally integrate the DSBH regions with the CRD, forming a compact functional module. This architecture ensures proper spatial alignment of cofactor-binding sites and catalytic residues, enabling the oxidative modification of methylated cytosines during demethylation 83, 84. Interestingly, Fu *et al.* reported that TETs could also participate in the dynamic and reversible modification of RNA cytosines 9. Multiple studies have indicated that TET1 and TET2 are required for the deposition of 5hmC in mRNA and tRNA and that TET-mediated 5hmC can reduce the stability of important pluripotency-promoting transcripts during embryonic stem cell (ESC) differentiation 9, 85-88. Importantly, a proteomic approach confirmed that TET1 and TET2 contain an RNA-binding domain 88. TET1-mediated m^5^C RNA modification, demethylation, and R-loop resolution during DNA repair are important for repair completion and the maintenance of genome stability 89. TET2 mutations with high frequency have been identified in multiple haematologic malignancies 90-93. The relationship between TET2 mutations and overall survival suggests that TET2 functions as a tumour suppressor 94, 95. For example, TET2 regulates the open state of active chromatin by oxidizing the m^5^C modification of caRNA and inhibits leukaemogenesis 96. Surprisingly, several findings on therapeutic resistance also support the tumour-promoting role of TET2 97-99.

ALKBH1, which is widely distributed in the cytoplasm, nucleus, and mitochondria, has substrate diversity and can remove multiple types of RNA modifications, such as N1-methyladenosine (m^1^A), m^6^A, m^5^C, and 3-methylcytidine (m^3^C) 100-102. It contains a central catalytic core, a nucleotide recognition lid (NRL) with Flip1 and Flip2, and a distinct N-terminal Flip0. In the catalytic core, there is a highly conserved DSBH structure 102. Notably, three unique structural features outside the core determine the high dependence of ALKBH1 on the secondary structure of the substrate. Specifically, ALKBH1 preferentially catalyses demethylation in bulged, bubbled DNA and various local unpaired nucleic acids (such as R-loops, stem loops, D-loops, and bulges) 103. Compared with TET2, ALKBH1 is the major m^5^C dioxygenase of RNA in human HEK293T cells, where it is responsible for the bulk of hm^5^C and f^5^C production 42. Hypoxia-induced ALKBH1 decreases the global m^5^C level in human extravasated trophoblast cells and can regulate mRNA stability 104. In addition, human ALKBH1 catalyses the hydroxylation and oxidization of m^5^C34 in both ct-tRNA^Leu^ and mt-tRNA^Met^, affecting mitochondrial translation and respiratory complex activity 104. To date, ALKBH1 has not been found to function as an RNA demethylase in malignant tumours.

### 2.3 Readers

Reader proteins, with special RNA-binding domains, are the ultimate executors of RNA methylation functions (Figure 2).

ALYREF, which is located mainly in the cell nucleus, is the first mRNA m^5^C-reading protein with the critical m^5^C recognition site K171 to be discovered, and it preferentially binds mature mRNA globally 10. As a component of the TREX complex, it facilitates the nuclear export of mRNA by specifically binding to mRNA with m^5^C modifications in the nucleus to form the mRNA-exporting protein (mRNP) complex 45, 105, 106. Mechanistically, CBP80 and PABPN1 are specifically involved in ALYREF recruitment to the 5′ and 3′ regions of mRNA. Moreover, CstF64 interacts with ALYREF and functions in ALYREF recruitment to the mRNA 105. Studies have suggested that ALYREF can play an essential role in metastasis, cancer progression, and chemoresistance by modulating cell proliferation, migration, and invasion and antiapoptotic effects 45, 107-110.

YBX1 is localized primarily in the cytoplasm and serves as an RNA m^5^C reader that plays a crucial role in regulating RNA metabolism 111. YBX1 comprises three primary structural domains: the cold shock domain (CSD), the alanine/proline domain (A/P domain), and the C-terminal domain (CTD) 112. These domains mediate complex interactions between YBX1 and both nucleic acids (DNA and RNA) and other proteins. The CSD uniquely contains nucleic acid-binding sites, enabling YBX1 to engage preferentially with m^5^C-modified RNA. High-resolution crystal structures have revealed that CSD interacts with RNA mainly through π-π stacking interactions assembled by four highly conserved aromatic residues (His-87, Phe-85, Phe-74, and Trp-65) 48, 113. Subsequently, it promotes mRNA stability in an m^5^C-dependent manner by recruiting the mRNA stabilizer ELAVL1 47. YBX1 is expressed in a broad range of tissues, and its roles in regulating cell proliferation, stress responses, and apoptosis make it crucial for normal development and tissue homeostasis 114, 115. In addition, its dysregulation has been linked to various diseases, including cancer 116-119.

## 3. The Fates of RNA Molecules with m^5^C Modifications

### 3.1 mRNA

Generally, m^5^C modification participates in four metabolic processes of mRNA, including pre-mRNA splicing, nuclear export, stability, and translation of mature mRNA, thereby altering the expression of m^5^C-related genes 10, 118, 120-122 (Figure 2). Taking the most common RNMP as an example, NSUN2 induces different biological mechanisms in various mRNAs. On the one hand, NSUN2 alters the methylation pattern of *PTEN* pre-mRNA, resulting in the downregulation of PTEN expression by mediating its alternative splicing events 123. On the other hand, NSUN2 functions as a writer of m^5^C modifications on *SRSF6* mRNA. The increasing level of m^5^C induce its nuclear‒cytoplasmic transport, which plays a vital role in multidrug resistance. In addition, NSUN2-mediated m^5^C modification enhances *FABP5* and *LAMC2* stability in osteosarcoma (OS) and head and neck squamous cell carcinoma (HNSCC), respectively 124, 125. Moreover, the overexpression of wild-type NSUN2 leads to gefitinib resistance and tumour recurrence, which are related to the m^5^C site at the CDS region of *QSOX1* mRNA. Interestingly, unlike others, the increasing m^5^C modification of *QSOX1* promotes its translation 126.

### 3.2 tRNA

tRNA shows the widest variety and largest number of RNA modifications, which are pivotal for stabilizing the tertiary structure of tRNA molecules (modifications outside of the anticodon loop) and decoding the genetic code (modifications in the anticodon loop). NSUN2 is upregulated in anaplastic thyroid cancer (ATC) and increases the m^5^C modification on tRNA^leu^ at the C48 site, which stabilizes tRNA^leu^ by preventing its cleavage. This stable tRNA^leu^ maintains homeostasis and rapidly transports leucine, substantially increasing the efficiency necessary to support the translation of c-MYC, BCL2, RAB31, JUNB, and TRAF2, among others. As pro-oncogenic proteins, they contribute to promoting tumour formation, proliferation, invasion, migration, and resistance to genotoxic drugs 127.

### 3.3 circRNA

circRNA is a class of covalently closed RNA molecules characterized by universality, diversity, stability and conservative evolution. Recent studies have shown that some epitranscriptomic modifications affect circRNA metabolism, such as stability, subcellular localization, and even translation. The m^5^C modification of *circFAM190B* increases its stability, which is dependent on NSUN2. *circFAM190B* targets SFN and regulates its ubiquitination, thereby inhibiting cellular autophagy through the SFN/mTOR/ULK1 pathway and ultimately promoting lung cancer development 128. The increased *circ_0102913* expression in cancer cells was attributed to NSUN5 at least partly because the hypermethylated m^5^C modification stabilizes the specific RNA. It subsequently enhances the malignant properties of cells via the *miR-571*/RAC2 axis 129. The carcinogenic effects of RAC2 might be attributed to its role in the alternative activation of macrophages 130. A combined m^5^C microarray analysis revealed that *circERI3* contains m^5^C modifications and that the NSUN4-mediated m^5^C modification of *circERI3* could increase its nuclear export. Additionally, *circERI3* inhibits DDB1 ubiquitination and regulates *PGC-1α* transcription through DDB1, thus increasing mitochondrial energy metabolism and ultimately contributing to the development of lung cancer 131.

### 3.4 lncRNA

lncRNA plays two distinct roles in epitranscriptomic modifications. On the one hand, lncRNA has emerged as a critical regulator of RMPs. In addition, there are many sites on their sequences that can be modified. In glioblastoma endothelial cells, NSUN2 increases the stability of *LINC00324* and upregulates its expression through m^5^C modification. *LINC00324* competes with the 3′-UTR of *CBX3* mRNA for binding to the AUH protein and reducing *CBX3* mRNA degradation. In addition, CBX3 directly binds to the promoter region of VEGFR2, enhancing VEGFR2 transcription and promoting angiogenesis 132. The stable lncRNA *NR_033928* with m^5^C modification can upregulate the expression of glutaminase by interacting with the IGF2BP3/HUR complex, which is a potential prognostic and therapeutic target in gastric cancer 133. The expression of *H19* lncRNA is abnormally increased in liver cancer, and this RNA is a specific target of NSUN2. Through m^5^C modification, its stability is significantly increased, and it recruits the oncoprotein G3BP1, further leading to the accumulation of MYC, which is a new mechanism of angiogenesis 134.

## 4. Functions and Mechanisms of m^5^C Modification in Cancer

**Figure 4:**
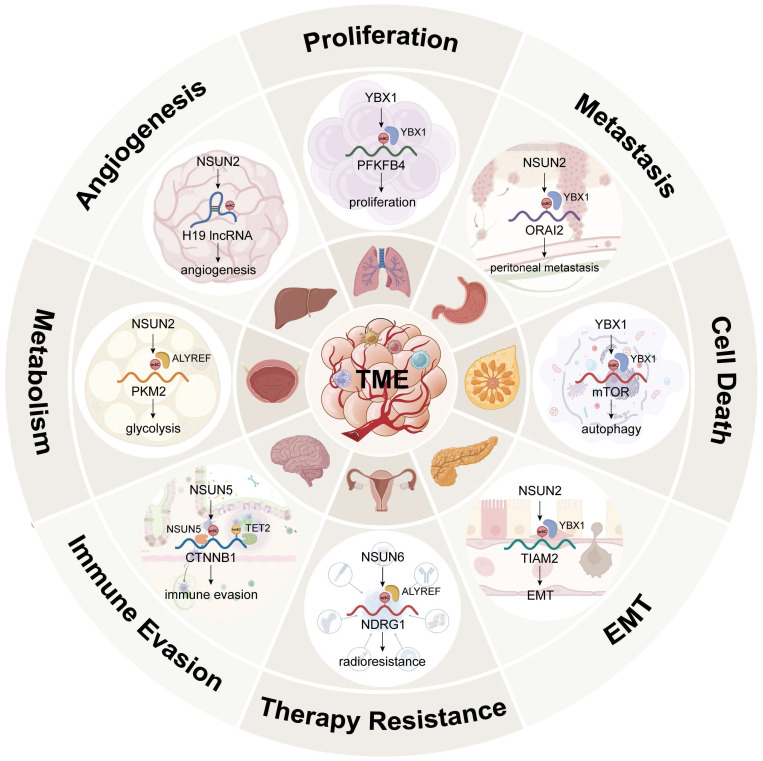
The functions and mechanisms of m^5^C in cancer. Created in BioRender. Mao, Z. (2025) https://BioRender.com/e9rc6z6.

To date, a total of 14 cancer hallmarks have been identified to explain the mechanisms of malignant tumour initiation, progression, and therapeutic response 135, 136. Among them, nonmutational epigenetic reprogramming, defined as enabling characteristics, was officially shown to play a significant role in 2022 137. Figure 4 and Table 1 summarize the functions and regulatory mechanisms of m^5^C in cancer.

### 4.1 Central nervous system cancers

#### 4.1.1 Glioma

The level of m^5^C modification in glioma tissue is significantly greater than that in peritumoral tissue and is positively correlated with the tumour grade 138. While NSUN2 and NSUN4 are highly expressed in glioma, single-cell bioinformatic analysis has revealed that malignant cells present the lowest NSUN5 expression levels among the different cell types that make up the tumour mass. NSUN2 methylates the 3′-UTR of *ATX* mRNA at the C2756 site in the human glioma cell line U87. With the recognition of ALYREF, *ATX* is exported from the nucleus to the cytoplasm and subsequently translated into ATX protein 139. ATX is a secreted glycoprotein that can convert lysophosphatidylcholine into lysophosphatidic acid (LPA), functioning as the major enzyme for extracellular LPA production. LPA can regulate a broad range of cell functions, such as cell survival, proliferation, and migration 140, 141. Malignant gliomas exhibit immune evasion characterized by increased expression of the immune checkpoint protein CD47 142. By combining databases, the m^5^C prediction website, and the MeRIP-qPCR assay, Zhao *et al.* revealed for the first time that NSUN4, as a key writer for controlling m^5^C levels in glioma, mediates changes in m^5^C levels to promote the stability of *CDC42* mRNA. This cascade, in turn, promotes activation of the PI3K-AKT pathway, culminating in the malignant progression of glioma cells 138. NSUN5 directly interacts with *CTNNB1* caRNA and increases its m^5^C modification, which is subsequently oxidized by TET2 to 5hmC. RBFOX2 functions as a 5hmC-specific reader to recognize and promote *CTNNB1* degradation. Finally, the downregulation of β-catenin interferes with the binding of CD47 to SIRPα, thereby weakening the phagocytosis of tumour-associated macrophages (TAMs) 143. Intriguingly, this study revealed that NSUN5 could act as an immune therapy target to transform glioma into a “warm tumour” and lead to impressive therapeutic outcomes when NSUN5 is restored in IDH1-R132H mutant glioma cells.

#### 3.1.2 Ocular cancer

The global and mRNA m^5^C levels are significantly enriched in retinoblastoma (RB) tissue compared with normal retinal tissue, which is attributed to the high expression of tumour-specific NSUN2 4. Through multiomic analysis, *PFAS* mRNA has been identified as a downstream candidate target of NSUN2. As a vital enzyme in purine biosynthesis, PFAS upregulated by m^5^C modification accelerates retinoblastoma progression, which bridges the current understanding of RNA modification and metabolic reprogramming. NSUN2 increases m^5^C modification on *CTNNB1* mRNA and then promotes uveal melanoma cell migration and proliferation by regulating the cell cycle 144.

### 4.2 Respiratory tract cancers

#### 4.2.1 Lung cancer

Lung cancer remains the leading cause of cancer-related deaths worldwide, and the most prevalent histological type is non-small cell lung cancer (NSCLC), which constitutes approximately 80% of all cases. NOP2, NSUN2, and NSUN4, key RNA m^5^C methyltransferases, are highly expressed in NSCLC tumour tissue, and their levels are strongly correlated with tumour grade, tumour size, and poor outcomes. In addition, ALYREF and YBX1, which are readers of m^5^C, are upregulated in lung cancer. However, the levels of NSUN6 are low in lung cancer, and NSUN6 may play a protective role. Cr(VI), a common environmental contaminant, has been shown to result in NSUN2 upregulation in human bronchial epithelial cells and mouse lung tissues 109. Using RNA-seq, MeRIP-seq, and MeRIP-qPCR, several targets of NSUN2 have been identified, including mRNAs (*QSOX1, NRF2, PD-L1, ME1, GLUT3,* and *CDK*) 109, 126, 145, 146 and circRNAs (*circFAM190B*) 128. Chen *et al.* reported that NSUN2-mediated m^5^C modification of the *NRF2* mRNA 5′-UTR enhances its stability in an m^5^C-YBX1-dependent manner. NRF2 is renowned for its integral role in managing ferroptosis, which relies on the disengagement of KEAP1 from NRF2 when faced with oxidative stress 145. Interestingly, the findings of m^5^C modification have shed light on a novel, noncanonical pathway in which NRF2 activation modulated by NSUN2 operates independently of the KEAP1-mediated mechanism. In contrast, NSUN2 posttranscriptionally enhances *PD-L1* mRNA stability, subsequently increasing PD-L1 expression in an m^5^C-ALYREF-dependent manner and providing protective effects for tumour cells against CD8^+^ T-cell-mediated cytotoxicity in NSCLC 146. Additionally, NOP2 and NSUN4 are highly expressed in lung cancer. The stable *EZH2* mRNA produced by NOP2 and ALYREF coregulation leads to EMT and promotes the malignant properties of cancer cells through the H3K27me3/E-cadherin axis 147. NSUN6 regulates NM23-H1 expression by modifying the 3′-UTR of *NM23-H1* mRNA through the m^5^C mechanism and inhibits cancer cell proliferation, migration, and EMT 148. These studies have greatly enriched the understanding of the role of writers other than NSUN2 in cancer regulation. The binding of ALYREF to *YAP1* mRNA inhibits the apoptosis of tumour cells through activation of the Hippo and Wnt/β-catenin pathways 149. YBX1 ensures the stability of *PFKFB4* mRNA by recognizing its 3′-UTR m^5^C sites in the cytoplasm after the exportation effect of THOC3 118. PFKFB4, a glycolysis regulator, produces pentose phosphate to perform carcinogenic functions 150.

#### 4.2.2 Nasopharyngeal carcinoma

Nasopharyngeal carcinoma (NPC) has high rates of metastasis and invasion, with a particularly high incidence in Southeast Asia, southern China, and North Africa 151, 152. NSUN2 and ALYREF are significantly upregulated in NPC tissues, and their high expression is correlated with poor distant metastasis-free survival (DMFS) and overall survival (OS) 107, 153. The analysis of GSEA RNA-seq data revealed that *NOTCH1* mRNA is m^5^C-modified by NSUN2. This protein is subsequently recognized and stabilized by ALYREF, which promotes NOTCH1 expression and activates the Notch signalling pathway in NPC cells. Notably, the evolutionarily conserved Notch signalling pathway plays an important role in determining the fate of NPC cells. Moreover, treatment with the NOTCH1 inhibitor LY3039478 and its relationship with prognosis in this study highlighted that ALYREF could serve as a therapeutic target and potential biomarker.

### 4.3 Digestive tract cancers

#### 4.3.1 Esophageal cancer

Esophageal squamous cell carcinoma (ESCC) is one of the most aggressive gastrointestinal malignancies worldwide, with a 5-year survival rate of approximately 20% 154, 155. The levels of RNA m^5^C methylation are substantially increased in ESCC tissues due to the upregulation of NSUN2 and NSUN5, which constitutes an important regulatory mechanism for ESCC progression 156-158. Additionally, ALYREF and YBX1 levels are also elevated in ESCC. NSUN2 increases the m^5^C modification on *GRB2* and *SMOX* mRNA and promotes their stability 156, 157. The upregulation of GRB2 evokes oncogenic PI3K/AKT and ERK/MAPK signalling 159. SMOX activates the mTORC1 signalling pathway with the recognition of YBX1. NSUN5 is also significantly upregulated in esophageal Cancer (EC) and shows promising diagnostic potential 158. Gene coexpression analysis of data from the databases GEPIA and UALCAN and site analysis from RMBase v3.0 have suggested that NSUN5 binds directly to the METTL1 transcript, facilitating its m^5^C modification in EC cells. METTL1, an m^7^G-modifying enzyme, has been identified as a novel epigenetic oncogene, and elevated METTL1 activity is essential for promoting EC tumour growth 160, 161. Oxaliplatin (L-OHP) is a potent chemotherapeutic agent that induces apoptosis in EC cells 162. However, its effectiveness is significantly hindered by the development of resistance. ALYREF expression is elevated in L-OHP-resistant EC tissues, and ALYREF further recognizes the m^5^C sites on *TBL1XR1* and *KMT2E* mRNAs, stabilizing these transcripts and promoting APOC1 expression 163. APOC1, a protransfer factor, plays a crucial role in the metabolism of very-low-density lipoprotein (VLDL) and high-density lipoprotein (HDL) cholesterol, predicting a poor prognosis and correlating with tumour immune infiltration 164-166. Within the cytoplasmic milieu of ESCC cells, *circPRKCA* interacts with YBX1, consequently preventing the ubiquitination-mediated degradation of YBX1. Increased concentrations of YBX1 increase the stability of *CSF2* mRNA in a m^5^C-dependent manner 167. CSF2, a tumour-derived growth factor, is widely recognized for its role in promoting angiogenesis, which is often a crucial process 168. Additionally, it drives EMT and enhances immune checkpoint protein expression, thereby facilitating the malignant progression of cancer 169, 170. Hence, these findings highlight the potential of RMPs as more comprehensive biomarkers due to the broader involvement of RMPs in the critical pathways and tumorigenesis of EC, providing a preclinical rationale for selectively targeting m^5^C modification as a promising therapeutic strategy.

#### 4.3.2 Gastric cancer

Gastric cancer (GC) is the fifth most common malignant tumour and the fourth leading cause of cancer-associated death worldwide 171. Overall, the RNA m^5^C content is increased in GC samples and is positively correlated with NSUN2 expression 5, 123, 133, 172-175. One reason for the upregulation of NSUN2 expression is that the SUMOylation of NSUN2 on the basis of SUMO-2/3 promotes its stability 5. In addition, studies have shown that the transcription factor E2F1 can activate NSUN2 expression via the AMPK pathway in a peritoneal high-fat environment. Increased NSUN2 regulates *ORAI2* mRNA stability through m^5^C modification via YBX1 recognition, thereby promoting ORAI2 expression and accelerating peritoneal metastasis via PI3K-AKT signalling in GC 173. Notably, in addition to being recognized by NSUN2, lncRNAs can also reversibly regulate NSUN2 expression and enrichment to further exert m^5^C-based functions in cells. For example, *FOXC2-AS1* increases the m^5^C methylation level of *FOXC2* mRNA by recruiting NSUN2, which is further recognized by YBX1 and regulates the proliferation, migration, and invasion of tumour cells 174. In addition, *DIAPH2-AS1* upregulates the expression of NSUN2 by stabilizing the NSUN2 protein and promotes the epitranscriptomic modification of *NTN1* mRNA in gastrointestinal cancer cells 172.

#### 4.3.3 Liver cancer

The main type of liver cancer is hepatocellular carcinoma (HCC), which is a primary malignant tumour originating from liver epithelial tissue or mesenchymal tissue 176. The overall m^5^C modification level and the levels of its RMPs, such as NOP2, NSUN2, ALYREF, and YBX1, are greater in HCC tissues than in adjacent tissues. ALYREF expression is significantly increased in HCC, and ALYREF can directly bind to and stabilize the m^5^C modification site in the 3′-UTR of *EGFR* mRNA. The subsequent activation of the STAT3 signalling pathway is a critical regulatory mechanism that mediates EMT 177. YBX1 is highly expressed in HCC and is associated with a poor prognosis. Analysis of RNA-seq and Ribo-seq data has revealed that RNF115 is the target of YBX1 in regulating HCC development 178. Mechanistically, YBX1 binds to the m^5^C site of the RNF115 mRNA 3′-UTR and interacts with EIF4A1 to bridge the 5′-UTR, promoting mRNA circularization and translation. RNF115, an E3 ligase, subsequently mediates K27 ubiquitination and autophagic degradation of DHODH to suppress ferroptosis. The main classic functions of m^5^C readers in cancer are stability and nuclear export. Interestingly, a new biological mechanism of YBX1 has been discovered in HCC. As a multitarget kinase inhibitor for Raf kinases, sorafenib has been approved as a first-line treatment for advanced HCC by the Food and Drug Administration (FDA) of the United States 179. Studies on m^5^C modification in HCC have revealed the role of NSUN2 and ALYREF in sorafenib resistance. By RNA-seq and RNA-BisSeq, several mRNAs, including *GRB2, RNF115, AATF, c-MYC, PKM2,* and* MALAT1*, have been identified as targets with abundant m^5^C sites 180-182. The enrichment of these mRNAs induces sorafenib resistance through various pathways, such as Ras signalling, glycolysis, and ferroptosis.

#### 4.3.4 Cholangiocarcinoma

Cholangiocarcinoma (CCA) is a significant contributor to cancer-related mortality, and its incidence is increasing on a global scale 183, 184. NSUN5 and ALYREF have been found to be upregulated in CCA tissues and cells 120, 185. A recent study has revealed that upregulated NSUN5 in CCA mediates the enrichment of glutaminase by increasing m^5^C modification at the cytosine 137 site within the untranslated region of *GLS* mRNA 186. Furthermore, GLS enhances cancer progression by impeding copper-induced cell death mechanisms. Copper is an essential trace element, and its homeostasis can impact cell metabolic processes and even confer resistance to chemotherapy 187. However, a surplus of copper leads to cuproptosis 188. These findings establish a correlation between m^5^C modification and cuproptosis in CCA for the first time, shedding light on the underlying molecular mechanisms and indicating a potential therapeutic target for this disease.

#### 4.3.5 Pancreatic cancer

Pancreatic cancer (PC) is one of the most lethal solid malignancies in which NSUN2, YBX1, and ALYREF are overexpressed. Notably, the highest incidence of perineural invasion (PNI) manifests mainly by the invasion of tumour cells into nerve tissue and their subsequent spread and metastasis along nerves 189, 190. The severity of PNI is associated with severe disease-related pain and poor survival 191. YBX1 enhances the stability of PNI-associated mRNAs, including *EGR1, NTRK1,* and* SMAD7*, through m^5^C modification 192. The increased secretion of migration inhibition factor (MIF) and tumour necrosis factor-α (TNF-α) promote invasion. Overall, epigenetic cross-talk between YBX1 and PNI in PC cells has been reported to be involved. YBX1 also affects the stability of *caspase-8* mRNA via m^5^C modification, resulting in increased caspase-8 expression and inhibition of RIPK1/RIPK3/MLKL pathway phosphorylation in PC 193. Overexpressed ALYREF might be a novel target for modulating pancreatic ductal adenocarcinoma (PDAC) metabolic vulnerability and immune surveillance 194. Investigations involving the JASPAR database and RNA-seq data have revealed that ALYREF specifically recognizes and stabilizes *JunD* mRNA, whose protein serves as a transcription factor of SLC7A5. As SLC7A5 is a key transporter of large neutral amino acids (LNAAs), the overexpression of SLC7A5 in tumour cells depletes amino acids in the TME and restricts the function of CD8+ T cells 195. In addition, the aberrant m^5^C modification mediated by NSUN2 in PC is associated with the upregulated expression of *TIAM2* mRNA, which promotes EMT and the likelihood of cancer cell migration 196.

#### 4.3.6 Colorectal cancer

In colorectal cancer (CRC), tissue immunohistochemistry has demonstrated an elevated level of m^5^C modification in tumour tissues compared with adjacent normal tissues. The m^5^C methyltransferases NSUN2, NSUN4, NSUN5 and the reader protein ALYREF exhibit significantly elevated expression and exert oncogenic functions 129, 197-200. By RNA-Seq and RNA-BisSeq, NSUN2 and YBX1 have been identified as "writers" and "readers" of *ENO1* and *SKIL* mRNAs in CRC cells. ENO1, the core catalytic enzyme of glycolysis, ultimately reprogrammes glucose metabolism and increases lactate production in an m^5^C-dependent manner. Interestingly, lactate accumulation in tumour cells, in turn, activates NSUN2 transcription via histone H3K18 lactylation (H3K18la) and induces NSUN2 lactylation at residue Lys356 (K356), which is essential for target RNA capture. The positive-feedback loop of the NSUN2/YBX1/m^5^C-ENO1 axis connects epigenetic remodelling and metabolic reprogramming 197. However, the elevated stability of *SKIL* mRNA ultimately increases transcriptional coactivator with PDZ-binding motif (TAZ) activation 198. As the first barrier of the body's defense, innate immunity plays a key role in tumour immune surveillance and anti-tumour response, in which type I interferon (IFN-I) is an important mediator with significant antiviral and anti-tumour functions 201-203. cGAS-STING signaling is a cytosolic DNA-sensing pathway that activates the expression of IFN-I 204, 205. In colon adenocarcinoma (COAD), GPX4 has emerged as the vital enzyme to prevent lipid peroxidation and maintain cellular redox homeostasis 206, 207. And NSUN5-mediated m^5^C modification on *GPX4* mRNA facilitated anticancer immunity via activation of cGAS-STING signaling by maintaining redox homeostasis 208, 209. Accumulating evidence has demonstrated the pivotal role of STING in the antitumour immune response, and the current receptor agonist exhibits potent antineoplastic activity in an immunocompetent mouse model of colon cancer 210. Therefore, m^5^C-regulated STING activation holds great potential for therapeutic intervention in cancer immunotherapy. Correlation analysis using the TCGA database and an RIP assay has revealed the direct binding of NSUN4 to *NXPH4* mRNA. By relying on the m^5^C-dependent mechanism, *NXPH4* mRNA can avoid degradation by RNautophagy. Furthermore, the competitive binding of the NXPH4 protein with PHD4 impedes HIF1A degradation and activates the HIF signalling pathway. Collectively, these results underscore a new regulatory pathway in which m^5^C-based NXPH4 plays a pivotal role in driving CRC progression 200. ALYREF is highly expressed in CRC tissues and predictive of a poor patient prognosis. Integrated analysis of the RIP-BisSeq and transcriptome profiles has revealed RPS6KB2 and RPTOR mRNAs as its downstream effectors. Additionally, ALYREF promotes tumour growth and migration by recruiting ELAVL1 to facilitate the nuclear export of these two transcripts 211.

### 4.4 Urinary system cancers

#### 4.4.1 Renal cell carcinoma

Clear cell renal cell carcinoma (ccRCC) patients are usually diagnosed at late stages 212. Therefore, it is imperative to find new strategies for ccRCC therapy. Excitingly, the overexpression of m^5^C RMPs, NOP2, and YBX1 has provided key insights into the treatment of solid ccRCC tumours 213. Several analyses, including analyses of TCGA transcriptome profiles, RNA-seq data, and BisSeq data, have revealed that *APOL1*, a participant in lipid transport and metabolism 214, is a downstream mRNA regulated by NOP2. YBX1 subsequently stabilizes *APOL1* mRNA by binding to the m^5^C site in the 3′-UTR, thus affecting ccRCC cell proliferation, migration, and invasion through the PI3K-Akt pathway. YBX1 also recognizes *PEBP1* mRNA via PEBP1P2 recruitment 215. PEBP1 is a crucial ferroptosis regulator that mediates many cancer-related processes, such as tumour development, metastasis, and the microenvironment 216, 217.

#### 4.4.2 Bladder cancer

m^5^C is frequently hypermethylated in urothelial carcinoma of the bladder (UCB) and enriched in oncogenic pathways, and NSUN2 and ALYREF have been found to be upregulated in these tissues. Interestingly, the aberrant expression of NSUN2 protein is partially attributed to high levels of m^5^C methylation of its mRNA 218. More specifically, ALYREF recognizes the hypermethylated m^5^C site of *NSUN2* mRNA, resulting in NSUN2 upregulation in UCB. BisSeq, RNA-seq, and RIP-seq analyses have revealed that elevated NSUN2 and ALYREF specifically bind to the m^5^C site in the target *TK1* and *RABL6* pre-mRNAs, contributing to splicing and stabilization. These results suggest a novel m^5^C-dependent mechanism of TK1 and RABL6 oncogene expression that enhances the proliferation and invasion of UCB cells. In addition, NSUN2 regulates the m^5^C site in the 3′-UTR of *HDGF* mRNA, and YBX1 controls its stability through the indole ring of W65 in its cold shock domain 47. As a well-known oncogene, HDGF is positively associated with aggressive UCB 219. HIF-1α induces transcriptional activation of ALYREF, which binds to m^5^C sites in the 3′-UTR of *PKM2* mRNA and enhances its stability 220. Hence, PKM2, a key enzyme in glycolysis, is upregulated and promotes the proliferation of cancer cells 221. These findings suggest that NSUN2, YBX1, and ALYREF play oncogenic roles in bladder cancer and participate in the complex regulatory network, providing new insights into the mechanisms of m^5^C modification in cancer.

#### 4.4.3 Prostate cancer

The m^5^C RNMTs NSUN2 and NSUN5 are expressed at higher levels in prostate cancer (PCa) tissues than in adjacent tissues. ACC1 is the first rate-limiting enzyme for fatty acid synthesis 222. Interestingly, phosphorylated NSUN5 increases the m^5^C modification on *ACC1* mRNA in PCa and enhances its stability and nuclear export with the recognition of ALYREF, thereby mediating CDK13-induced lipid accumulation and synthesis to promote PCa growth 223. These findings indicate that a previously unrecognized m^5^C-based CDK13-NSUN5-ACC1 axis mediates fatty acid synthesis and lipid accumulation in PCa cells. Lipid metabolism is an extremely important metabolic change in the TME of PCa 224, 225. NSUN2 expression is also upregulated in PCa and is associated with a poor prognosis 226. Epitranscriptome assays with RNA-BisSeq analysis have revealed that the 5′-end regions of *AR* mRNA are modified by NSUN2 and stabilized by an m^5^C-YBX1-dependent mechanism, which influences several AR variants, including AR-V7. AR is one of the most crucial therapeutic targets in PCa 227, 228. Since 2012, several new AR inhibitors, such as enzalutamide, abiraterone, and apalutamide, have been approved to treat castration-resistant PCa 229. However, stimulation of AR variants by AR inhibitors could induce drug resistance because of self-activation without androgen binding 230. The positive feedback between NSUN2 and AR provides novel evidence that m^5^C modification clusters exist in PCa and explain cancer progression and the occurrence of castration-resistant PCa.

### 4.5 Gynaecological cancers

#### 4.5.1 Cervical cancer

Radiotherapy is the main treatment for advanced cervical cancer (CC) 231. The level of m^5^C modification is greater in patients with radioresistance, which is related to overexpression of NSUN6 m^5^C protein and associated with a poor prognosis. Integration of MeRIP-seq and mRNA-seq analysis has revealed that *NDGR1* is a downstream target mRNA of NSUN6 and that its stability is increased by specific binding to the m^5^C reader ALYREF. Abnormal overexpression of NDGR1 promotes homologous recombination (HR)-mediated DNA damage repair (DDR) by recruiting and stabilizing the HR-related protein RDA51, which leads to radiotherapy resistance in CC 232. Additionally, NSUN2 is also upregulated in CC, increases m^5^C modification on *LRRC8A* and *KRT13* mRNA, and promotes tumour cell migration and invasion via the YBX1 reader 233, 234. When cancer cells swell, volume-regulated anion channels (VRACs) are activated 235, 236. LRRC8A has recently been identified as an essential component of VRACs that can promote the proliferation and migration of cancer cells in CC. Although the role of KRT13 is different in distinct cancers depending on the context 237, 238, research has revealed that the NSUN2-YBX1-KRT13 pathway stimulates CC cell migration and invasion.

#### 4.5.2 Endometrial cancer

Endometrial cancer (EC), the incidence of which has increased by more than 50% during the past two decades, is the most common cancer within the female reproductive system in developed countries 171. NSUN2 and YBX1 are significantly overexpressed in EC 239. BisSeq analysis of mRNAs derived from HEC-1B cells has revealed enrichment of ferroptosis-related pathways among differentially methylated genes. Furthermore, NSUN2 promotes *SLC7A11* mRNA stability via the recognition role of YBX1, which resists the ferroptosis pathway of tumour cells to promote survival. These results provide new insight into the mechanisms of m^5^C-based ferroptosis regulation and suggest a promising treatment strategy for EC patients.

#### 4.5.3 Ovarian cancer

Ovarian cancer (OC) has the highest death rate and the worst prognosis of all gynaecological tumours 240, and cytoreductive surgery combined with chemotherapy remains the gold standard of treatment 241. However, chemotherapy resistance followed by intraperitoneal dissemination still leads to unpredictable deaths. YBX1, an m^5^C reader, is highly expressed and maintains the stability of various mRNAs, including *CDH3, E2F5, YY1,* and* RCC2,* by recognizing their m^5^C sites, which ultimately leads to drug resistance in cancer cells 242, 243. Notably, CHD3, an important member of the chromodomain helicase DNA-binding protein (CHD) family, which is involved in regulating chromatin remodelling 244, 245, is a key protein in the response of YBX1 to stress induced by platinum-based drugs. Specifically, highly expressed CHD3 promotes chromatin opening and further enhances HR repair by HR-related proteins such as BRCA1 and RAD50. Platinum resistance is the primary barrier affecting the prognosis of OC patients; hence, the working model of the m^5^C-CDH3-chromatin accessibility-HR repair axis proposed by these researchers is important for developing therapies that can reverse platinum resistance. In addition, the presence of NOP2 and NSUN2 in OC is associated with the hypermethylation of *RAPGEF4* and *E2F1* mRNA, respectively, leading to uncontrolled proliferation, migration, and invasion of tumour cells 246, 247.

#### 4.5.4 Breast cancer

Breast cancer (BC) poses a significant threat to women's health because of its intricate pathogenesis and diverse clinical manifestations 248, 249. Notably, the absolute number of BC cases is increasing in many developing countries due to population growth and the adoption of Western lifestyles 250. Studies show that most m^5^C RMPs are significantly dysregulated in BC tissue, and regulate tumorigenesis, progression, prognosis, drug resistance and immune landscape 251. The malignant phenotype of BC is partially promoted by the overexpression of NSUN2 and YBX1. Through a combination of RNA-Seq and RNA-BisSeq, *HGH1* has been identified as a target RNA of NSUN2 252. YBX1 synergistically regulates the expression of HGH1 in an m^5^C-dependent manner by increasing its RNA stability and overall protein synthesis efficiency. The role of HGH1 in human physiology and pathology has rarely been reported. These results preliminarily clarify the biological role that HGH1 might play in the progression of BC. The findings of the m^5^C mechanism in *HGH1* mRNA also reveal a regulatory pathway from posttranscriptional modification to protein translation. Additionally, YBX1, which is stably mediated by SAT1, recognizes the m^5^C modification site of *mTOR* mRNA and significantly inhibits autophagy through this gatekeeper of the *mTOR* signalling pathway in triple-negative BC (TNBC) 253. The involvement of m^5^C modification in TNBC, the most aggressive subtype with the poorest prognosis, reveal the complicated interaction between autophagy and tumour progression.

## 5. Clinical Implications of m^5^C Modification in Cancer

**Figure 5:**
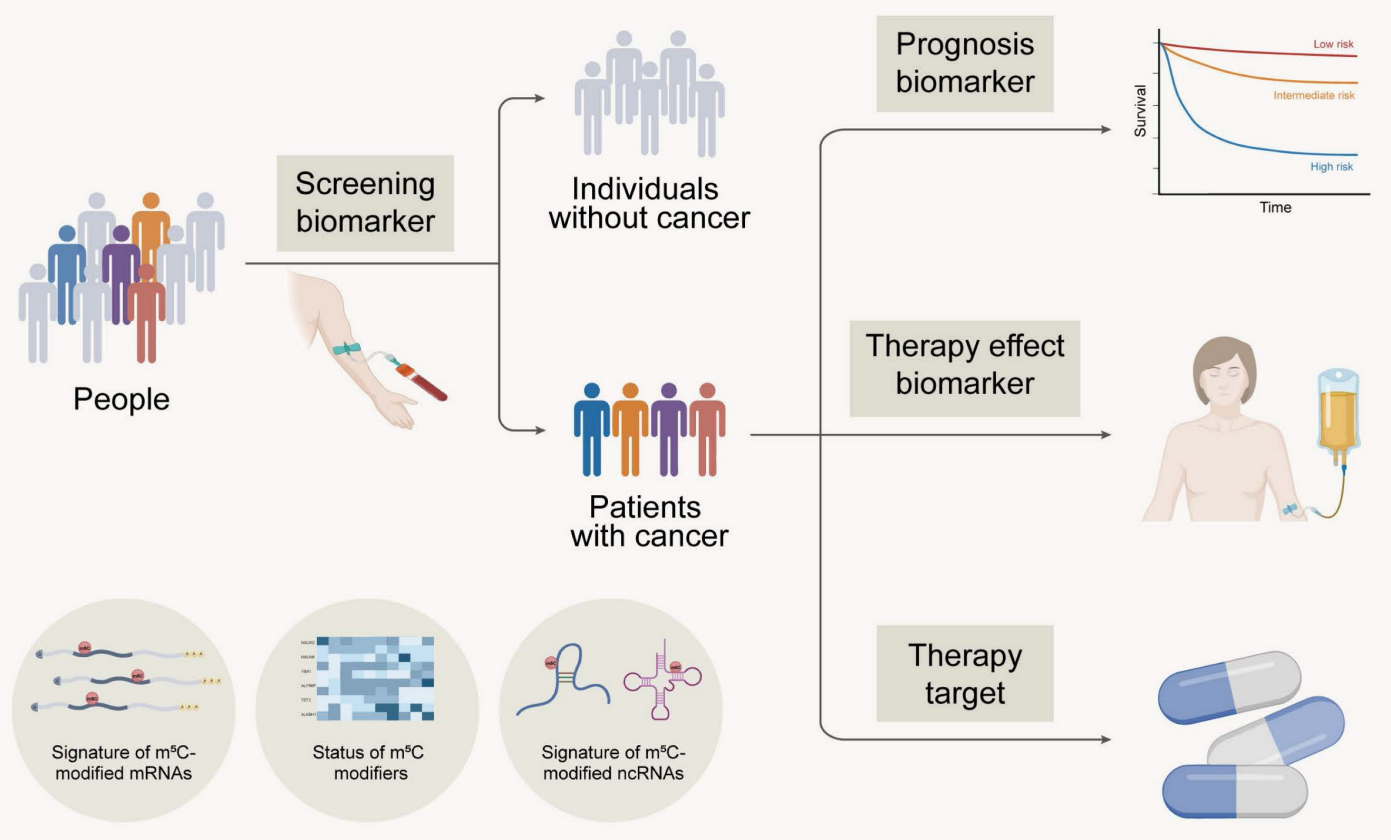
Clinical implications of m^5^C modification in cancer. Created in BioRender. Mao, Z. (2025) https://BioRender.com/49digcl.

Recently, the profiles and signatures of m^5^C in RNA, including the expression and mutation of m^5^C proteins and the m^5^C modification levels of mRNA and ncRNA, are closely related to the clinical characteristics of patients with tumours. These findings suggest that m^5^C, as a potential biomarker and therapeutic target, is expected to be applied in clinical practice to benefit cancer patients (Figure 5).

### 5.1 m^5^C as a biological marker

Technical advances over the past two decades, especially the unprecedented progression of next-generation sequencing (NGS) technology, have enabled robust diagnosis and detection of cancer in biological samples. In addition to tissue biopsy, liquid-based biopsy assays have been proposed, with a focus on biomarkers, circulating tumour cells (CTCs) 254, circulating tumour DNA (ctDNA) 255, tumour-induced extracellular vehicles (EVs) 256, and other components in body fluids such as blood, urine, and saliva. The level of m^5^C modification and the status of m^5^C RMPs are associated with tumorigenesis. Yin *et al.* reported that the m^5^C level in peripheral blood immune cells was significantly increased in patients with colorectal cancer and that the degree of m^5^C modification was positively correlated with tumour progression and metastasis. Therefore, m^5^C methylation in peripheral blood immune cells is a promising biomarker for noninvasive diagnosis 257.

The clinical and pathological characteristics of tumours, such as stage, pathological type, and treatment sensitivity, determine the prognosis of patients. Increasing evidence has shown that m^5^C plays an important role in cancer 258. Therefore, m^5^C-related features have become a powerful tool for predicting patient prognosis. Huang *et al.* constructed a survival prediction model for patients with TNBC on the basis of the mRNA expression profiles of NSUN6 and NSUN2 in the TCGA database. The risk score of each patient was calculated using the following formula: risk score = -0.5714 × NSUN6 + 0.024 × NSUN2, where NSUN6 is a protective factor, and NSUN2 is a risk factor. The prediction model shows good performance in evaluating the overall survival (OS) of patients in the public database 259. According to the TCGA data, genetic alterations in endogenous m^5^C RMPs were observed in 236 out of 297 CC patients (79%) 260. This high prevalence underscores the translational potential of these alterations as promising diagnostic biomarkers and therapeutic targets. Based on consistent clustering map of 13 m^5^C RMPs, upregulation of NSUN2, NSUN3, NSUN6, and TET2, coupled with the downregulation of NSUN5 and ALYREF, is associated with poor survival outcomes of CC patients. Besides, a 4-gene m^5^C signature comprising FNDC3A, VEGFA, OPN3, and CPE has also demonstrated remarkable 1-year, 3-years and 5-years prognostic capabilities 261. This refined understanding of m^5^C RMPs and gene signatures enables the development of a novel molecular diagnostic test, facilitating prognostic assessment and the identification of potential therapeutic targets for CC patients. By integrating mRNA expression data from TCGA, GEO, and real-world cohorts, Liu *et al.* successfully identified 6 candidate m^5^C-related genes (*SOCS2, LCAT, FTCD, KRT17, PBK*, and *CBX2*) and constructed an m^5^C scoring model that can be used to effectively predict the prognosis of patients. Survival analysis in the real-world cohort (2^-△△CT^-based risk score) revealed that the prognostic risk score model was a strong independent prognostic factor.

Treatment resistance in cancer is challenging for doctors regarding the decision-making process in clinical practice. For example, pancreatic ductal adenocarcinoma is the most aggressive malignant tumour of the digestive tract and is highly resistant to treatment. Duo *et al.* used unsupervised consensus clustering analyses, LASSO, and multivariate Cox regression analysis to construct an m^5^C scoring signature (m^5^C score). They reported that the m^5^C score was associated with the activation of cancer-related pathways, including the Ras, MAPK, and PI3K pathways. Therefore, the sensitivity of patients to pathway-specific inhibitors of PARP, EGFR, AKT, HER2, and mTOR could be evaluated to guide the use of targeted drugs 262.

### 5.2 m^5^C as a therapeutic target

Tumour-targeted therapy, also known as molecular-targeted drug therapy, refers to drugs or biological products that inhibit tumour growth and development in local tumour tissue. Such approaches can reduce the toxic effects on normal cells by inhibiting the key signalling pathways involved in tumour initiation and progression and provide more precise and effective strategies. NSUN2 expression is significantly increased in CRC and plays a carcinogenic role. Chen *et al.* identified a biologically active small-molecule inhibitor in the ChemDiv library that could effectively inhibit NSUN2 expression. The NSUN2 inhibitor NSUN2-i4 significantly enhances the efficacy of PD-1 against colorectal cancer without causing significant toxicity, indicating that NSUN2 is has promise as a target for cancer immunotherapy combined with an immune checkpoint inhibitor (ICI) 197. Research has revealed that NSUN2 is upregulated in AML and that the inhibition of NSUN2 prevents AML progression* in vivo* in xenograft experiments 263. These results indicate that targeting NSUN2 may offer new strategies for treating AML. SU056 is an azodiamidazole-like small molecule that efficiently inhibits the function of the YBX1 protein. Recent studies have shown that targeting YBX1 is expected to reverse platinum resistance in ovarian cancer 242. 5-Fluorouracil (5-FU) is a first-line chemotherapeutic agent for advanced GC 264. YBX1 is significantly upregulated in 5-FU-resistant GC cell lines and patient tissues, and YBX1 knockdown increases apoptosis in resistant cells treated with 5-FU 265. These findings establish YBX1 as a key regulator of autophagy and 5-FU resistance in GC and highlight its potential as a novel therapeutic target for overcoming 5-FU resistance.

## 6. Conclusion and Future Perspectives

Lifestyle changes, increased access to early screening, and improved treatment continue to reduce cancer-related mortality. However, the incidence of malignant tumours such as those of breast, prostate, and endometrial cancer continues to increase annually. The morbidity of cervical and colorectal cancer tends to be greater in younger patients, which causes serious economic and social burdens around the world. Recently, with advances in technology, in-depth research in the field of epitranscriptomics has revealed the critical role of m^5^C RNA modification in regulating many cellular pathways 122, 192, 266-268. However, its potential functions in cancer have not been fully explored. To date, m^5^C modification is common in rRNA 269, but evidence that rRNA m^5^C modification regulates reprogramming in cancer is currently lacking. Whether there are more m^5^C regulatory proteins requires further analysis and demonstration 270, 271. Moreover, whether RMPs exhibit selectivity or complementarity for the m^5^C modification sites of RNA is worth further investigation. In particular, why does m^5^C modification occur in specific mRNAs during cancer progression? We postulate that the m^5^C modification has a stoichiometric effect. In general, numerous RNAs undergo chemical modification to varying degrees, resulting in a dynamic and reversible equilibrium process. However, during the initiation, progression, and treatment of malignant tumours, dysregulation of RMPs elevates RNA modification levels beyond a critical dose threshold. This disruption breaks the dynamic equilibrium state, rendering it irreversible. Although current research cannot systematically explain the substrate specificity of m^5^C modification, we propose the following three hypotheses: high CG content, high transcriptome abundance, and structural accessibility. First, specific RNAs possess a high CG content, making them more readily recognizable by RNMTs. Additionally, some RNAs constitute a relatively large proportion of the overall transcriptome, consequently increasing the probability of modification events. Furthermore, the structural conformation of these RNAs renders potential m^5^C sites more exposed, thereby increasing their accessibility to catalytic enzymes. Hence, a more detailed examination of the regulatory patterns of m^5^C modification in different parts of a single transcript is essential for advancing our understanding of pathophysiological processes. The small molecules NSUN2-i4 and SU056, which are NSUN2 and YBX1 inhibitors, have been demonstrated to enhance the efficacy of immunotherapy and chemotherapy in mouse models. However, the development of drugs that target m^5^C modification is still a long way off. Owing to the success of mRNA vaccines in the prevention and treatment of infectious diseases, we are interested in the use of mRNA vaccines in the context of cancer immunotherapy 272. Whether m^5^C modification could be applied to mRNA vaccine development deserves further consideration. In addition, in almost all types of malignant tumours, the overall level of m^5^C modification is elevated, which is related to the deposition of writer proteins in tumour cells. Generally, the factors influencing the expression of writers include genomic mutations and environmental changes. For example, persistent high-risk HPV infection can interfere with the expression of RNMPs at the DNA, RNA and protein levels in cervical cancer. Understanding the upstream regulatory elements of RNMPs can provide valuable insights for clarifying the origin of cancer, developing screening methods, and preventing cancer. In summary, the characteristics, mechanisms, and potential application value of m^5^C modification in cancer need further exploration.

## Figures and Tables

**Table 1:** The functions and mechanisms of m^5^C RMPs in cancer.

Type	RMPs			Target RNA		Mechanism	Function	Ref.
Glioma	Writer	NSUN2	Up	ATX	Nuclear export, Translation (ALYREF)	ATX-LPA axis	Migration	139
				LINC00324	Stability	CBX3-VEGFR2 axis	Proliferation, Migration	132
		NSUN4	Up	CDC42	Stability (ALYREF)	PI3K-AKT signaling	Proliferation, Migration, Invasion	138
		NSUN5	Down	CTNNB1	Stability	TET2-RBFOX2 axis	Immune evasion	143
HNSCC	Writer	NSUN2	Up	LAMC2	Stability (YBX1)	-	Proliferation, Migration, Invasion, EMT	125
Retinoblastoma	Writer	NSUN2	Up	PFAS	Stability (ALYREF)	-	Proliferation	4
Nasopharyngeal carcinoma	Reader	ALYREF	Up	NOTCH1	Stability (ALYREF)	Notch signaling	Proliferation, Migration, Invasion	153
Thyroid cancer	Writer	NSUN2	Up	SRSF6	Nuclear export (ALYREF)	UAP1-AGX2-ABC transporter axis	Multidrug resistance	108
				tRNA^Leu^	Stability	c-MYC/BCL2/RAB31/JUNB/TRAF2	Proliferation, Migration, Invasion, Chemotherapy resistance	127
Esophageal cancer	Writer	NSUN2	Up	GRB2	Stability	PI3K-AKT and ERK/MAPK signaling	Proliferation, Migration, Invasion	157
				SMOX	Stability (YBX1)	mTORC1 signaling	Proliferation, Migration, Invasion	156
				NLRP3	Nuclear export, Stability (ALYREF, YBX1)	NLRP3/caspase 1/IL-1β inflammatory pathway	Proliferation, Migration, Invasion	121
		NSUN5	Up	METTL1	-	-	Proliferation	158
	Reader	ALYREF	Up	TBL1XR1, KMT2E	Stability	Upregulate APOC1 expression	Oxaliplatin resistance	163
		YBX1	Up	CSF2	Stability	-	Migration, Invasion, Glycolysis	167
Breast cancer	Writer	NSUN2	Up	HGH1	Stability, Translation (YBX1)	Bind to EEF2	Proliferation, Migration, Invasion	252
	Reader	YBX1	Up	mTOR	Stability	-	Proliferation, Migration, Autophagy	253
Lung cancer	Writer	NOP2	Up	EZH2	Stability (ALYREF)	H3K27me3-E-cadherin axis	Migration, Invasion, EMT	147
		NSUN2	Up	QSOX1	Translation (YBX1)	-	EGFR-TKIs resistance	126
				NRF2	Stability (YBX1)	Enhance the transcription of GPX4, FTH1, and other antioxidants	Proliferation, Migration, Invasion, Ferroptosis	145
				PD-L1	Stability (ALYREF)	Inhibit CD8+ T-cell infiltration	Immune evasion	146
				CircFAM190B	Stability	SFN-mMOR-ULK1 axis	Proliferation, Migration, Apoptosis	128
				ME1, GLUT3, CDK2	Stability (ALYREF)	-	Proliferation, Migration, Invasion, Angiogenesis, Cell cycle, Metabolism	109
		NSUN4	Up	CircERI3	Nuclear export	DDB1-PGC-1α-mitochondria axis	Mitochondrial energy metabolism, Proliferation, Migration, Cell cycle, Apoptosis	131
				CDC20	Stability (ALYREF)	-	Proliferation, Migration, Invasion	110
		NSUN6	Down	NM23-H1	-	-	Proliferation, Migration, EMT	148
	Reader	ALYREF	Up	YAP1	Stability	Hippo and Wnt/β-catenin signaling	Proliferation, Migration, Invasion, Apoptosis, Cell cycle, Therapy resistance	149
		YBX1	Up	PFKFB4	Stability	-	Proliferation, Migration, Glycolysis	118
Gastric cancer	Writer	NSUN2	Up	NR_033928	Stability	HUR/IGF2BP3-GLS axis	Proliferation, Apoptosis, Glutamine metabolism	133
				NTN1	Stability	-	Migration, Invasion, Neural invasion	172
				ORAI2	Stability (YBX1)	PI3K-AKT signaling	Proliferation, Migration, Invasion, Peritoneal metastasis	173
				PIK3R1, PCYT1A	-	-	Proliferation, Migration, Invasion, Chemotherapy resistance	5
				FOXC2	Stability (YBX1)	-	Proliferation, Migration, Invasion	174
				PTEN	Splicing	PI3K-AKT signaling	Proliferation, Migration	123
				ATG9A	Stability (YBX1)	-	5-Fluorouracil resistance, Autophagy	265
Liver cancer	Writer	NOP2	Up	c-MYC	Stability, Translation	LDHA/PKM2/ENO1/TPI1	Glycolysis	180
		NSUN2	Up	GRB2, RNF115, AATF	-	Ras signaling	Sorafenib resistance	181
				H19	Stability	Recruit the G3BP1 oncoprotein	Proliferation, Migration, Invasion, Angiogenesis	134
				SREBP2	Stability (YBX1)	-	Proliferation, Migration, EMT, Cholesterol metabolism	119
				PKM2	Stability	-	Proliferation, Migration, Glycolysis	182
				MALAT1	Stability (ALYREF)	ELAVL1-SLC7A11 axis	Ferroptosis, Sorafenib resistance	267
				SOAT2	Stability	-	Proliferation, Migration, Invasion, Apoptosis, Immune evasion	58
	Reader	ALYREF	Up	EGFR	Stability	STAT3 signaling	Proliferation, Migration, Invasion, EMT	177
		YBX1	Up	RNF115	Circularization, Translation	DHODH K27 ubiquitination	Ferroptosis	178
Cholangiocarcinoma	Writer	NSUN5	Up	GLS	Stability	-	Proliferation, Migration, Invasion, Cuproptosis	186
	Reader	ALYREF	Up	PKM2	Stability	-	Proliferation, Migration, Glycolysis, Ferroptosis	120
Pancreatic cancer	Writer	NSUN2	Up	TIAM2	Stability (YBX1)	-	Proliferation, Migration, Invasion, EMT	196
	Reader	YBX1	Up	EGR1, NTRK1, SMAD7	Stability	MIF/TNF-α	Perineural invasion	192
				Caspase-8	Stability	PIPK1/PIPK3/MLKL pathway	Proliferation	193
		ALYREF	Up	JunD	Stability	SLC7A5-mTORC1 signaling	Proliferation, Immune evasion	194
Colorectal cancer	Writer	NSUN2	Up	ENO1	Stability (YBX1)	-	Proliferation, Invasion, Glycolysis	197
				SKIL	Stability (YBX1)	Activate TAZ expression	Proliferation, Migration	198
				SLC7A11	Translation, Stability	-	Proliferation, Ferroptosis	199
				KSR1	Stability (YBX1)	ERK/MAPK signaling	Migration, Invasion	59
		NSUN4	Up	NXPH4	Stability	PHD4-HIF1A axis	Proliferation, Migration, Invasion, RNautophagy	200
		NSUN5	Up	circ0102913	Stability	miR-571-RAC2 axis	Proliferation, Migration, Invasion	129
				GPX4	Stability	cGAS-STING signaling	Anticancer immunity	208
	Reader	ALYREF	Up	RPS6KB2, RPTOR	Nuclear export	-	Proliferation, Migration	211
Renal cancer	Writer	NOP2	Up	APOL1	Stability (YBX1)	PI3K-AKT signaling	Proliferation, Migration, Invasion	213
	Reader	YBX1	Up	PEBP1	Stability	-	Migration, Invasion	215
Bladder cancer	Writer	NSUN2	Up	RABL6, TK1	Splicing, Stability (ALYREF)	-	Proliferation, Invasion	218
				HDGF	Stability (YBX1)	-	Proliferation, Migration, Invasion	47
	Reader	ALYREF	Up	PKM2	Stability	-	Glycolysis	220
Prostate cancer	Writer	NSUN2	Up	AR	Stability (YBX1)	-	Proliferation, Migration, Invasion	226
				TRIM28	Stability	-	Proliferation, Migration	60
		NSUN5	Up	ACC1	Nuclear export (ALYREF)	-	Proliferation, Lipid deposition	223
Ovarian cancer	Writer	NOP2	Up	RAPGEF4	-	-	Proliferation, Migration, Invasion	247
		NSUN2	Up	E2F1	Stability (YBX1)	MYBL2/RAD54L	Proliferation, Migration, Invasion	246
	Reader	YBX1	Up	CDH3	Stability	HR-related proteins, such as BRCA1, RAD50, NBS1, RAD51, etc.	Apoptosis, Cisplatin resistance	242
				E2F5, YY1, RCC2	Stability	-	Proliferation, Migration, Invasion, Chemoresistance	243
Endometrial cancer	Writer	NSUN2	Up	SLC7A11	Stability (YBX1)	-	Ferroptosis resistance	239
Cervical cancer	Writer	NSUN2	Up	LRRC8A	Stability (YBX1)	PI3K-AKT signaling	Proliferation, Migration, Invasion	233
				KRT13	Stability (YBX1)		Migration, Invasion	234
		NSUN6	Up	NDRG1	Stability (ALYREF)	HR-mediated DNA damage repair	Radioresistance	232
Leukemia	Writer	NSUN2	Up	PHHGH, SHMT2	Stability (YBX1)	-	Proliferation, Apoptosis, Serine metabolism	263
	Eraser	TET2	Down	TSPAN13	Stability (YBX1)	-	stem cell homing and self-renewal	93
Melanoma	Writer	NSUN2	Up	CTNNB1	-	c-MYC/Cyclin D1	Proliferation, Migration	144
	Reader	YBX1	Up	MAGEA1	Stability	P53 signaling	Stemness, Proliferation, Migration, Invasion	268
Osteosarcoma	Writer	NSUN2	Up	FABP5	Stability	-	Proliferation, Migration, Invasion, Fatty acid metabolism	124
		NSUN6	Up	EEF1A2	Stability	AKT/mTOR signaling	Proliferation, Migration, Invasion	122

## Abbreviations

m^5^C: 5-methylcytidine
BisSeq: bisulphite sequencing
ncRNA: noncoding RNA
RMPs: RNA-modifying proteins
SAM: S-adenosyl-methionine
CDS: the coding sequence
MeRIP-seq: methylated RNA immunoprecipitation sequencing
NSUN: NOL1/NOP2/SUN
DNMT: DNA methyltransferase
ALKBH1: alkB homologue 1
ALYREF: Aly/REF export factor
YBX1: Y-box binding protein 1
RNMTs: RNA methyltransferases
TRD: target recognition domain
TRED: target recognition extension domain
PARPis: PARP inhibitors
TC-HR: transcription coupled-homologous recombination
Alt-NHEJ: alternative nonhomologous end joining
DSB: double-strand break
VL: variable loop
SSU: small subunit
LSU: large subunit
CRD: cysteine-rich domain
DSBH: double-stranded β-helix
ESC: embryonic stem cell
NRL: nucleotide recognition lid
CSD: cold shock domain
CTD: C-terminal Domain
OS: osteosarcoma
HNSCC: head and neck squamous cell carcinoma
EMT: epithelial-mesenchymal transition
LSCs: leukemia stem cells
BM: bone marrow
ATC: anaplastic thyroid cancer
LPA: lysophosphatidic acid
TAMs: tumour-associated macrophages
RB: retinoblastoma
NSCLC: non-small cell lung cancer
NPC: nasopharyngeal carcinoma
DMFS: distant metastasis-free survival
OS: overall survival
ESCC: esophageal squamous cell carcinoma
L-OHP: oxaliplatin
VLDL: low-density lipoprotein
HDL: high-density lipoprotein
GC: gastric cancer
HCC: hepatocellular carcinoma
FDA: the Food and Drug Administration
CCA: cholangiocarcinoma
PC: pancreatic cancer
PNI: perineural invasion
MIF: migration inhibition factor
TNF-α: tumour necrosis factor-α
PDAC: pancreatic ductal adenocarcinoma
LNAAs: large neutral amino acids
CRC: colorectal cancer
TAZ: PDZ-binding motif
IFN-I: type I interferon
COAD: colon adenocarcinoma
ccRCC: clear cell renal cell carcinoma
UCB: urothelial carcinoma of the bladder
PCa: prostate cancer
CC: cervical cancer
HR: homologous recombination
DDR: DNA damage repair
VRAC: volume-regulated anion channel
EC: endometrial cancer
OC: ovarian cancer
CHD: DNA-binding protein
BC: breast cancer
TNBC: triple-negative breast cancer
NGS: next-generation sequencing
CTCs: circulating tumour cells
ctDNA: circulating tumour DNA
EVs: extracellular vehicles
ICB: immune checkpoint inhibitor
